# The Action Mechanism of the Myc Inhibitor Termed Omomyc May Give Clues on How to Target Myc for Cancer Therapy

**DOI:** 10.1371/journal.pone.0022284

**Published:** 2011-07-21

**Authors:** Mauro Savino, Daniela Annibali, Nicoletta Carucci, Emilia Favuzzi, Michael D. Cole, Gerard I. Evan, Laura Soucek, Sergio Nasi

**Affiliations:** 1 Consiglio Nazionale delle Ricerche - Istituto di Biologia e Patologia Molecolari (CNR – IBPM), Dipartimento di Biologia e Biotecnologie, Università Sapienza, Roma, Italia; 2 Department of Molecular Biology, Princeton University, Princeton, New Jersey, United States of America; 3 Department of Pathology, University of California San Francisco (UCSF), San Francisco, California, United States of America; 4 Vall d'Hebron Institute of Oncology (VHIO), Barcelona, Spain; Institute of Genetics and Molecular and Cellular Biology, France

## Abstract

Recent evidence points to Myc – a multifaceted bHLHZip transcription factor deregulated in the majority of human cancers – as a priority target for therapy. How to target Myc is less clear, given its involvement in a variety of key functions in healthy cells. Here we report on the action mechanism of the Myc interfering molecule termed Omomyc, which demonstrated astounding therapeutic efficacy in transgenic mouse cancer models *in vivo*. Omomyc action is different from the one that can be obtained by gene knockout or RNA interference, approaches designed to block all functions of a gene product. This molecule – instead – appears to cause an edge-specific perturbation that destroys some protein interactions of the Myc node and keeps others intact, with the result of reshaping the Myc transcriptome. Omomyc selectively targets Myc protein interactions: it binds c- and N-Myc, Max and Miz-1, but does not bind Mad or select HLH proteins. Specifically, it prevents Myc binding to promoter E-boxes and transactivation of target genes while retaining Miz-1 dependent binding to promoters and transrepression. This is accompanied by broad epigenetic changes such as decreased acetylation and increased methylation at H3 lysine 9. In the presence of Omomyc, the Myc interactome is channeled to repression and its activity appears to switch from a pro-oncogenic to a tumor suppressive one. Given the extraordinary therapeutic impact of Omomyc in animal models, these data suggest that successfully targeting Myc for cancer therapy might require a similar twofold action, in order to prevent Myc/Max binding to E-boxes and, at the same time, keep repressing genes that would be repressed by Myc.

## Introduction

Myc transcriptional regulators – c-, N- and L-Myc – are basic, helix-loop-helix, leucine zipper (bHLHZip) proteins that bind to an array of genomic sites to either induce or repress transcription of genes important for cell growth, metabolism and differentiation [1]–[4]. These factors – especially c-Myc and N-Myc – are deregulated in the majority of human cancers. This is usually not due to *Myc* gene mutation but results from upstream mutations affecting other oncogenes or tumor suppressors. Myc activity thus appears to be required for development and maintenance of the majority of tumors, even when initiated by other causes. In tumors induced by Myc upregulation in transgenic mice, even brief Myc de-activation triggers tumor regression accompanied by growth arrest, differentiation, and collapse of the tumor vascular system [5]. Myc operates within a highly interconnected interactome network and a possible strategy for targeting Myc oncogenic function is dominant interference of Myc protein interactions. In this respect, Myc oligomerization domain – the bHLHZip region – proved to be capable of dominantly inhibiting Myc transforming ability in rat embryo fibroblast cells [6], [7]. This domain mediates the direct interaction with Max and sequence specific binding to specific consensus sequences – the E boxes – in promoters of activated target genes. The bHLHZip domain is also involved in the interaction with other partners – such as Miz-1 – that mediate transcriptional repression by Myc [4], [8]. To interfere with Myc protein-protein interactions, we developed a dominant negative molecule by introducing four selected mutations in the bHLHZip region of human c-Myc. The resulting 90 amino acid miniprotein – termed Omomyc for its capacity to form homodimers – is able to inhibit c-Myc/Max association, to affect transcriptional activation by c-Myc, and to enhance c-Myc dependent apoptosis in tissue culture cells [9], [10]. It was shown to prevent c-Myc induced papillomatosis *in vivo* without affecting tissue homeostasis [11] suggesting a capacity of targeting tumor cells without damaging the normal tissue.

Despite its pervasive role in human cancer, Myc met with considerable skepticism as a therapeutic target since its requirement for proliferation and maintenance of adult stem cell compartments raised concern about the toxicity of Myc inhibition for healthy tissues [12], [13]. In part thanks to work on Omomyc the potential of Myc as a therapeutic target is now established. Many doubts about selectivity for tumors have been dispelled by studies showing that transiently inhibiting Myc in skin, intestinal epithelium and other tissues does not dramatically alter tissue homeostasis [11], [14], [15]. The efficacy and safety of Myc targeting *à la Omomyc* has been conclusively demonstrated by reversible expression of Omomyc in transgenic models [16], [17]. Systemic expression of Omomyc attenuated proliferation in rapidly dividing tissues, but this was well-tolerated; tissue homeostasis was maintained, no apoptosis was observed in the normal tissues and all side effects were readily reversed following Omomyc removal. In Ras-driven lung tumors, the impact of Omomyc was impressive. Mice continuously expressing Omomyc failed to develop lung adenocarcinoma. In mice that had previously developed advanced cancer, induction of Omomyc triggered tumor regression which was accompanied by reduced proliferation and increased apoptosis of the tumor tissue [16]. An analogous anticancer impact was found in a simian virus 40 (SV40)-driven pancreatic islet tumor model [17], in breast cancer (G. Evan, personal communication) and glioma (in preparation). So, manipulating Myc function similarly to Omomyc might have the potential of an effective anticancer strategy for various tumor types.

In view of the striking properties of this molecule, it is extremely relevant to elucidate its mechanism of action. Omomyc biological effects are simply the results of *tout court* Myc function ablation, as it would occur with Myc gene or mRNA knockouts? Clearly, to explain the remarkable properties of Omomyc it is important to understand whether they result from selective targeting of the Myc interactome and how they impact on the Myc activated and repressed targets. These issues are addressed in the present work.

Our data indicate that Omomyc does not cause a global inhibition of Myc function but acts as an edge-specific perturbation of the Myc interactome, channeling its activity towards transrepression. This may be key to its success as an anticancer agent.

## Results

### Omomyc selectively targets the Myc interactome

**Figure 1:**
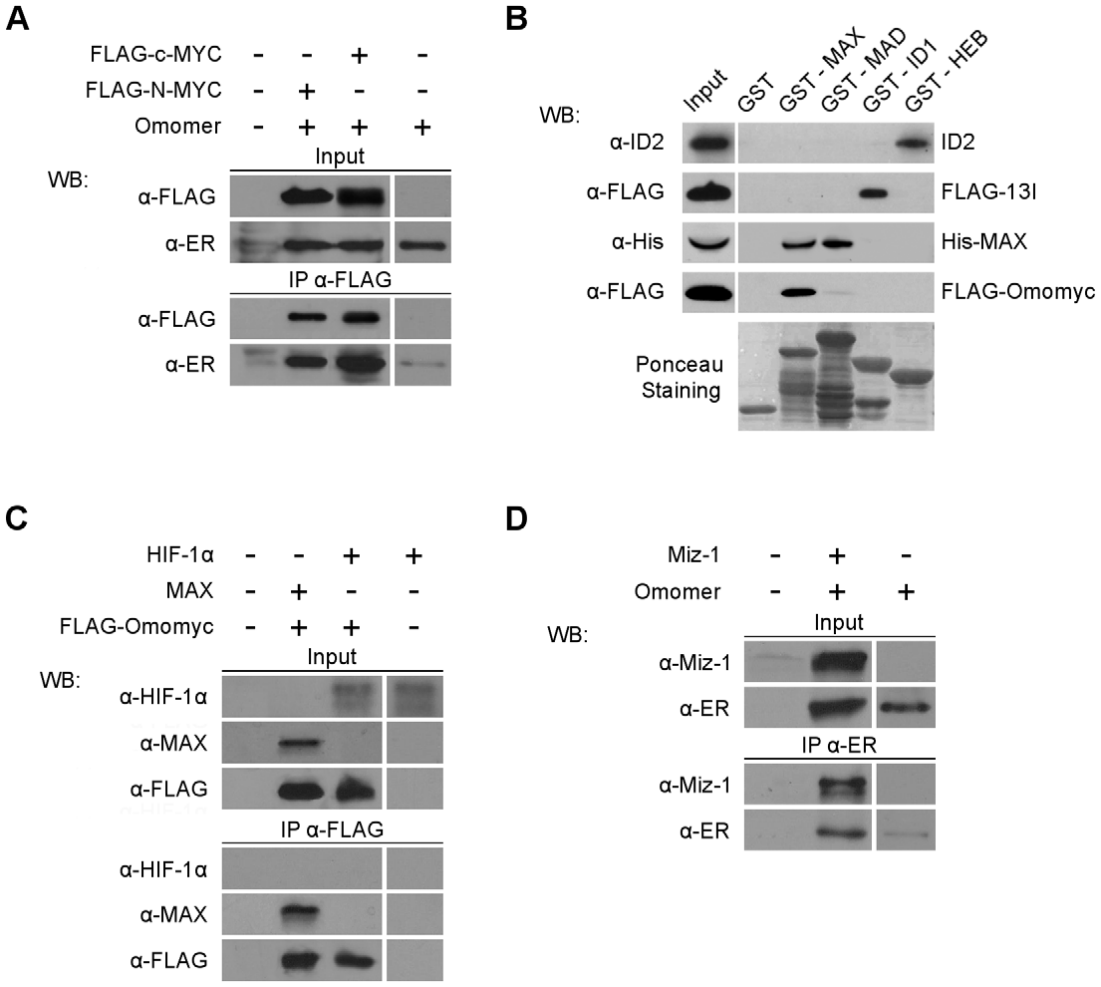
Omomyc binding specificity. A) **Omomyc binds c-Myc and N-Myc.** Immunoblotting (WB) with FLAG and ER antibodies – as indicated – of immunoprecipitations performed with FLAG antibodies on 293T cells transfected with FLAG-c-Myc or FLAG-N-Myc expressing vectors together with the Omomer expressing vector. B) **Omomyc binds Max but not Mad and two representative HLH proteins.** Immunoblotting (WB) with FLAG antibodies of GST pull-down assays performed with GST, GST-MAX, GST-MAD, GST-ID1 and GST-HEB (10 ìg each) on 293T cells transfected with the FLAG-Omomyc expressing vector. 293T cells transfected with ID2 or FLAG-13I [65] expressing vectors were used as positive control for GST-HEB and GST-ID1, respectively. Extracts from bacteria expressing His-MAX were used as positive controls for GST-MAX and GST-MAD. C) **Omomyc does not bind Hif-1α.** Immunoblotting (WB) with Hif-1α, Max and FLAG antibodies – as indicated – of immunoprecipitations performed with FLAG antibodies on 293T cells transfected with Hif-1α, Max and FLAG-Omomyc expressing vectors. D) **Omomyc binds Miz-1.** Immunoblotting (WB) with Miz-1 and ER antibodies – as indicated – of immunoprecipitations performed with Miz-1 antibodies on 293T cells cotransfected with Miz-1 and Omomer expressing vectors.

Direct physical interaction with the bHLHZip protein Max is crucial to Myc function: the Myc/Max complex binds DNA – recognizing E-boxes – and works as a transcriptional activator [18]. Omomyc is able to homodimerize, to form heterodimers with c-Myc and Max proteins, and to interfere with c-Myc/Max complex formation and binding to E-boxes in vitro [9], [10]. To better address Omomyc selectivity we investigated its capacity to bind N-Myc – which shares substantial functional redundancy with c-Myc and has important roles in tumor formation in the nervous system –, Mad – a strictly related bHLHZip factor that dimerizes with Max, binds E-boxes and acts as transcriptional repressor [4] –, Heb and Id1 – two HLH proteins representative of a large family of transcriptional regulators implicated in developmental processes [19]. To assess the capacity to bind N-Myc we performed immunoprecipitations on 293T cells ectopically expressing Omomyc fused to the oestrogen receptor ER™ – Omomer [10] – together with FLAG tagged c- or N-Myc. We found that Omomyc bound to N-Myc similarly to c-Myc (Figure 1A), in agreement with the virtual identity of the bHLHZip domain amino acid sequences of Myc family proteins. To assess binding to Max, Mad and the two HLH proteins Heb (an E protein) and Id1 we performed pull-down assays with GST-linked Max, Mad, Heb and Id1 on extracts of 293T cells ectopically expressing FLAG-tagged Omomyc. Whereas binding to Max was strong as previously reported [9], Omomyc binding to Mad was barely visible and binding to Heb and Id1 was undetectable (Figure 1B). The faint signal in the GST-Mad pull-down is likely due to the very high levels of FLAG-Omomyc expression in the transfected cells and not reflective of physiological interaction between the two proteins. In summary, we found that Omomyc binding specificity for Max and Mad proteins was the same as Myc. Similarly to Myc, Omomyc does not appear to interact with HLH proteins and therefore does not act by disrupting HLH protein networks crucial for differentiation control [20]–[22]. We then asked wether Omomyc interacted with Hypoxia-inducible factor 1-alpha (Hif-1α), a protein that functions as a master transcriptional regulator of the adaptive response to hypoxia, plays an essential role in tumor angiogenesis and shows a considerable interplay with Myc in the regulation of multiple glycolytic genes [23]–[25]. Hif-1α contains a bHLH domain and antagonizes Myc by binding to Max [24]. To investigate Omomyc binding to Hif-1α we performed immunoprecipitations on 293T cells ectopically expressing FLAG tagged Omomyc together with Hif-1α. We found (Fig. 1C) that Omomyc does not bind Hif-1α, while Max does as reported. Altogether, these experiments clearly indicate that Omomyc is Myc-Max-Mad network specific and – within this network – selectively affects Myc/Max dimerization, required for Myc binding to E-boxes and transactivation of a large number of genes.

**Figure 2:**
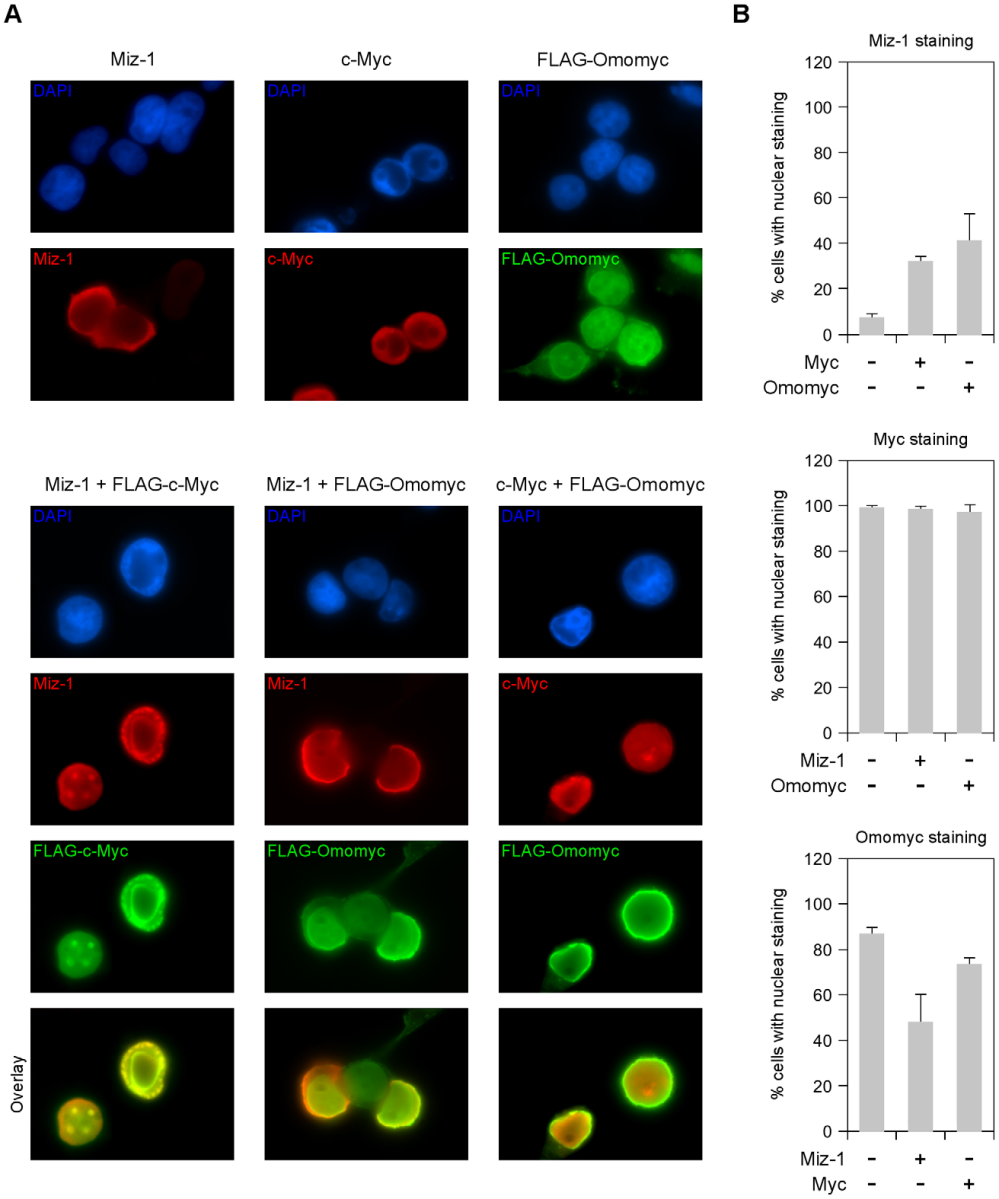
Omomyc intracellular co-localization with Miz-1 and c-Myc. A) Immunofluorescence photomicrographs of 293T cells transfected with Miz-1, c-Myc, FLAG-c-Myc and FLAG-Omomyc expressing plasmids - as indicated above panels - and stained with antibodies against FLAG (green), Miz-1 (red) and c-Myc (red) as indicated inside panels. Nuclei of the same fields were stained in blue with DAPI (4,6-diamidino-2-phenylindole). B) Quantitation of the experiments shown in A), based on the examination of 15 microscopy field and at least three biological replicates.

A key aspect of Myc function is transrepression of numerous genes involved in growth control, differentiation and tumor suppression [4]. This activity does not appear to implicate direct Myc binding to E-boxes. Myc is recruited to promoters of repressed genes only indirectly, upon interaction with proteins that directly bind to such promoters. The best known of them is Miz-1, a zinc finger protein involved in Myc dependent repression of cell cycle inhibitors – p15-INK4b (Cyclin-dependent kinase 4 inhibitor B) and p21(CDKN1: cyclin-dependent kinase inhibitor 1) – and in Myc dependent apoptosis in response to growth factor withdrawal [26]–[29]. Presumably in complex with Max, Myc contacts Miz-1 N-terminal region through the amino acid sites D394 and S405 [28] that are located in the HLH region. As these sites are not mutated in Omomyc, we hypothesized that Omomyc retained the capability to bind Miz-1. We tested this possibility via immunoprecipitation on 293T cells ectopically expressing Omomer and Miz-1. Figure 1D demonstrates that Omomyc directly interacts with Miz-1. Endogenous Miz-1 was reported to accumulate in the cytoplasm of cells, with a minor fraction in the nucleus; Myc overexpression triggers nuclear translocation of Miz-1 and sequestration in discrete subnuclear foci [30]. To investigate the interaction and intracellular localization of Omomyc, Omomyc/Miz-1 and Omomyc/c-Myc complexes, we transfected 293T cells with FLAG tagged Omomyc or c-Myc expressing plasmids together with plasmids expressing untagged Miz-1 and c-Myc, and detected protein localization by immunofluorescence with anti FLAG, c-Myc, and Miz-1 antibodies (Figure 2). In cells transfected with single expression plasmids, Miz-1 was mostly (about 90%) localized in the cytoplasm as previously reported [30], Myc was present exclusively in the nucleus as expected, and Omomyc was largely in the nucleus (about 85%) with a minor part in the cytoplasm (Figure 2). Omomyc lacks the nuclear localization signal of Myc proteins: it may enter the nucleus due to its small size or upon dimerization with endogenous Myc or Max. When co-transfected, most Myc proteins co-localized with Omomyc in the nucleus; a fraction of Omomyc remained in the cytoplasm - not associated to Myc - likely due to a higher level of expression. Ectopic Myc expression triggered nuclear translocation of Miz-1 (about 40%) and formation of discrete subnuclear foci, as previously reported [30]. Miz-1 was partly relocated in the nucleus in the presence of an overexpressed Omomyc as well (about 35%). Endogenous Miz-1 – which is present in low amounts within cells [30] – will presumably be mostly in the nucleus in the presence of an overexpressed Myc or Omomyc.

Altogether, these findings show that perturbation of the Myc interactome by Omomyc results not only from the inhibition of Myc binding to Max – which is required for E-box binding and transactivation – but also from the interaction of Omomyc with Miz-1, a Myc binding partner involved in transrepression.

### Omomyc affects the transcriptional response of fibroblasts to serum stimulation

**Figure 3:**
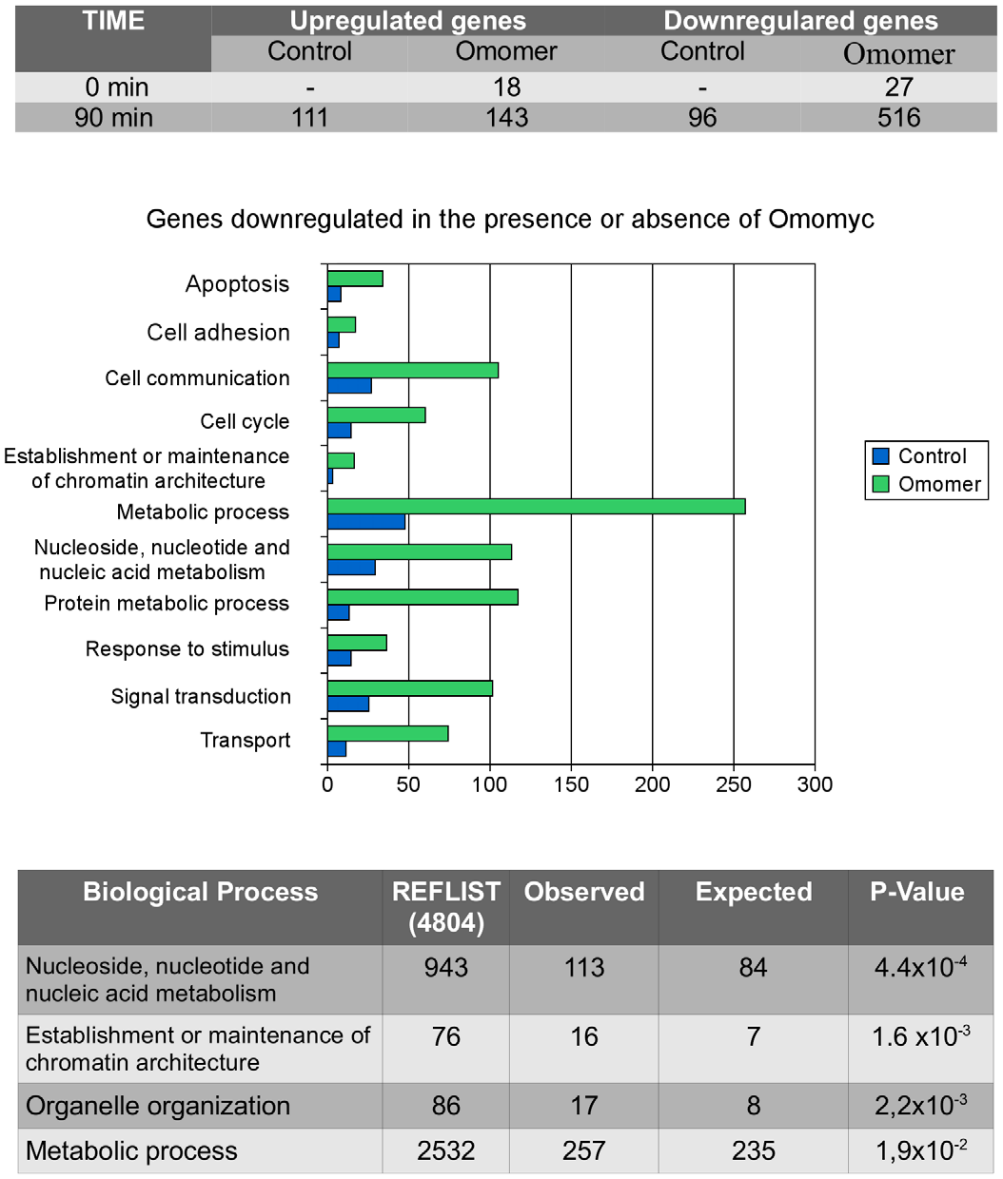
Omomyc impact on the transcriptional response of Rat1 fibroblasts to serum stimulation. Omomyc promotes downregulation of numerous genes. Top. Many more genes were downregulated in Rat1 cells expressing Omomer (Omomer) than in cells that do not (Control) at 90′ following serum stimulation of Rat1 fibroblasts in the presence of tamoxifen. The number of upregulated genes was similar. Rat1-control cells at time 0 were taken as reference. Middle. Distribution in different Biological Process classes – based on Gene Ontology classification – of genes downregulated at 90′ following serum stimulation in Omomer and control cells. Bottom. Genes related to nucleotide and nucleic acid metabolism are the most significantly over-represented – as computed by the Panther software – among the genes downregulated in the presence of Omomyc (Omomer cells in the presence of tamoxifen).

**Figure 4:**
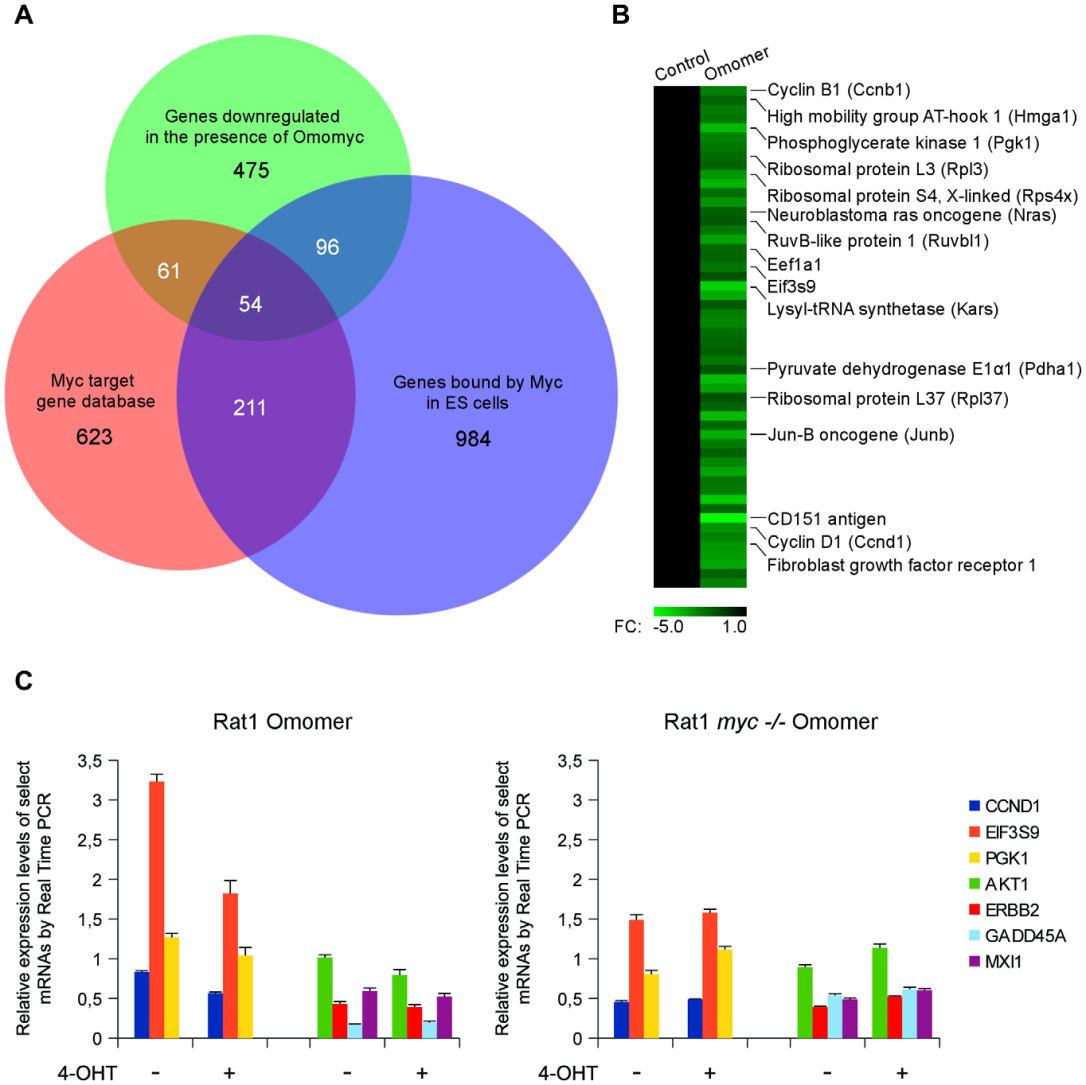
Upregulation of Myc activated targets is compromised whereas downregulation of Myc repressed targets is supported by Omomyc. A) Venn diagram illustrating the overlap among genes downregulated by Omomyc in the present study, genes listed as Myc targets in the Myc target gene database (www.myccancergene.org), and genes reported to be directly bound by c-Myc in ES cells [29], [38]. Within the Myc target gene database and the ES cell datasets, only genes that had a probe on the Affymetrix U34A array were taken into account. B) Overlapping, bona fide Myc target genes were extracted from our dataset and clustered in one dimension. C) Relative expression level (Fold Change) – measured by Real Time PCR at 90 min following serum stimulation in the presence or absence of 4-OHT – of *Ccnd1*, *Eif3s9*, *Pgk1*, *Akt1*, *Erbb2*, *Gadd45a*, *Mxi1* mRNAs in wild type (left) and Myc null (right) Rat1 cells expressing tamoxifen inducible Omomyc (Omomer). *Ccnd1*, *Eif3s9*, *Pgk1* represent Myc activated targets; *Akt1*, *Erbb2*, *Gadd45a*, *Mxi1* represent Myc repressed targets. Expression at the time of serum addition (time 0) was taken as reference for calculating the Fold Change.

Myc proteins are able to bind to 10–20% of genomic loci and to modulate expression of hundreds to thousands of genes. Omomyc was shown to impair transactivation of the Myc activated target gene *cad* in Rat1 fibroblasts and not to affect downregulation of the Myc repressed target *gadd45* [10]. To get an insight into widespread gene expression changes influenced by Omomyc, we analyzed the transcriptional response to serum stimulation of Rat1 fibroblasts stably infected with an Omomer producing or an empty retrovirus (Rat1-Omomer and Rat1-control cells, respectively, as described in [10]). c-Myc is known to be downregulated by serum starvation and sharply induced by serum addition – together with a number of other immediate early genes – reaching a peak after 1 to 2 h; c-Myc induction has a role in cell cycle re-entry [31]–[33]. Apart from cell proliferation, the serum response of fibroblasts integrates other processes – e. g. wound healing [34] – and serum regulated genes include genes that are regulated by Myc as well as genes that are not. Cells were serum-starved before serum stimulation and mRNA expression was analyzed at the time of serum re-addition (time 0) and 90′ thereafter – a time point at which Myc induction is maximal – so that induction of a direct Myc target should closely follow the expression of Myc. mRNA expression was analyzed via an oligonucleotide array containing probes for approximately 7,000 full-length sequences and 1,000 EST (expressed sequence tags) clusters. Data are accessible in the GEO database with the following accession number: GSE25039. Relative mRNA expression values (Fold Changes) were computed by taking as reference Rat1-control cells at time 0; only genes showing a Fold Change of at least +3 (upregulated genes) or −3 (downregulated genes) were taken into account. At the time of serum stimulation, Omomer expressing and control Rat1 cells displayed a virtually identical gene expression profile (Figure 3, top). At 90 min of serum induction 111 sequences were upregulated and 96 downregulated in Rat1-control cells. Strikingly, a pronounced downregulation was found in Rat1-Omomer cells: as many of 516 gene sequences were downregulated and 143 upregulated (Table S1). Overall, the total number of sequences whose expression was up or downregulated in the presence of Omomyc after 90 min of serum stimulation amounted to 8.2% of the probes on the array. Among the sequences downregulated in the presence of Omomyc, 475 represented genes with a known GeneID (gene identifier) and 41 other transcribed regions. To find out whether genes downregulated in the presence of Omomyc were also validated Myc targets we compared them to the set of genes listed in the Myc Cancer Gene database (www.myccancergene.org), a collection of Myc responsive genes identified in independent studies through a variety of techniques [35]. 115 (24%) of the 475 genes downregulated in the presence of Omomyc were listed in the Myc target gene database: 45 were listed as upregulated and 35 as downregulated by Myc, while the up or down regulation of the remaining 35 genes was unknown. To point out direct Myc targets within the list of genes downregulated in the presence of Omomyc, the list was crossed with those from two studies that used ChIP analysis to define genome wide promoter occupancy by Myc in embryonic stem (ES) cells [36], [37]. 31.5% of the genes downregulated in the presence of Omomyc had promoters that were reported to be directly bound by Myc in ES cells (P-value: 1.3×10^−24^) as compared to 19% in its absence (P-value: 3×10^−2^). Altogether, 44.4% of the Omomyc downregulated genes turned out to be genuine Myc targets according to either the Myc target genes database or the listing of Myc bound promoters in ES cells (Venn diagram: Figure 4A). Genes common to all three groups are clustered in one dimension in Figure 4B. Such a large – albeit incomplete – overlap among genes whose downregulation was associated to Omomer activation in Rat1 fibroblasts and validated Myc targets is highly significant. That the overlap is incomplete is consistent with other analyses of expression and computationally derived gene sets [38], [39], and it might be due in part to other effects of serum. Figure 3 (middle) shows the classification in functional categories based on GO (Gene Ontology) terms. As reported for genes associated with c-Myc activity [36], [40], [41], genes affected by Omomyc fall into multiple functional classes involving growth, metabolism, cell signaling pathways, cell cycle progression, and apoptosis. Notably, genes downregulated in the presence of Omomyc were enriched in categories – nucleotide and nucleic acid metabolism – that are overrepresented among targets upregulated by c-Myc [36], [40]–[42], supporting the notion of Omomyc as an inhibitor of Myc mediated transactivation (Figure 3, bottom). Targets known to be upregulated by Myc and found to be downregulated by Omomyc include genes encoding proteins directly involved in translation and ribosome assembly – such as the translation initiation factor Eif3s9, the translation elongation factor Eef1a1, and the ribosomal protein L3 and S4 – as well as genes encoding metabolic enzymes – isocitrate dehydrogenase 2, lactate dehydrogenase B and phosphoglycerate kinase 1 –, proteins involved in cell cycle progression – the anaphase-promoting complex subunit Anapc5, Cyclin B1 and Cyclin D1 –, the ATPase/DNA helicase TIP49 (Ruvbl1), and the structural chromatin protein Hmgb1. Hmgb1 has an important role in chromatin remodeling and is a mediator of inflammation whose overexpression is associated with many tumor types [43]. TIP49 is an important Myc cofactor, involved in Myc transcriptional and oncogenic functions [44]. Several genes found to be downregulated by Omomyc and known to be Myc repressed targets encode proteins involved in signaling pathways, cell adhesion and transcription – such as Akt1, Erbb2, c-Jun and the fibroblast growth factor receptor Fgfr1 – while *Mxi1* encodes a protein that interacts with Max and negatively regulates Myc function. *Mxi1* is repressed by Myc [45] but activated by Hif-1α [46], [47]. Defects in this gene have been reported in prostate cancer and in a subset of glioblastomas [48], [49].

To further validate our findings, we performed Real Time PCR assays in wild type and Myc null Rat1 fibroblasts expressing tamoxifen inducible Omomyc (Figure 4C). We compared – at 0 and 90 min of serum stimulation – the expression levels of a select sample of genes affected by serum stimulation that are known to be either repressed – *Gadd45a*, *Mxi1*, *Erbb2*, *Akt1* – or activated – *Pgk1*, *Eif3s9*, *Ccnd1* – by Myc [40], [45], [50]. These genes, like any gene, are not exclusive Myc targets, and other transcription factors modulated or not by serum may contribute to their regulation. We found that the expression levels of the repressed targets *Gadd45a*, *Mxi1*, *Erbb2* and *Akt1* in wild type Rat1 cells were unaffected or even more repressed in the presence of Omomyc whereas upregulation of the activated targets *Pgk1*, *Eif3s9* and *Ccnd1* was compromised (Figure 4C, left). In the Myc null cells, we found that Omomyc did not significantly affect expression of the repressed and did not impair upregulation of the activated targets (Figure 4C, right). Therefore the effect of Omomyc on transcription of Myc activated targets appears to require some activity of Myc.

In sum, these findings indicate that Omomyc does not globally inhibit Myc function in transcriptional regulation of target genes and suggest that it selectively perturbs the Myc transcriptome by hampering Myc mediated transactivation and preserving Myc mediated repression.

### Omomyc differentially affects transactivation and repression by influencing Myc binding to target gene promoters

**Figure 5:**
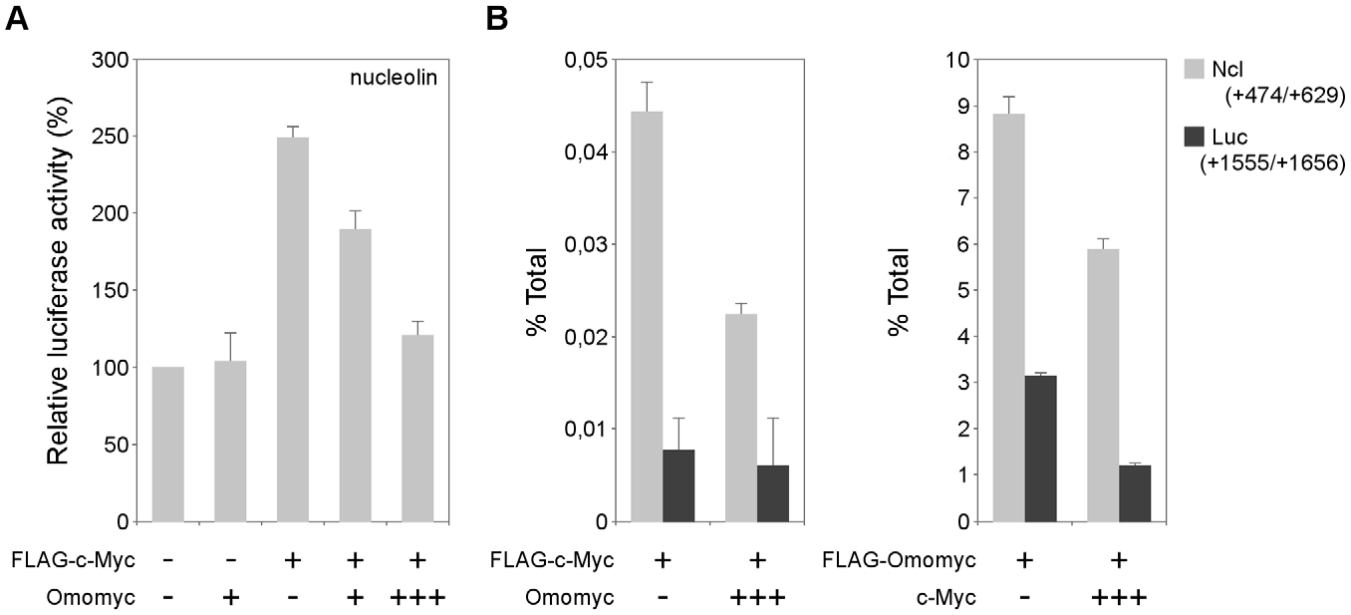
Omomyc inhibits Myc binding and transactivation of the *nucleolin* promoter. A) Luciferase activity of the mouse *nucleolin* promoter reporter plasmid – pNucL14 – transfected in 293T cells together with FLAG-c-Myc and Omomyc expressing vectors. Data were normalized by cotransfection of pRL-TK Renilla luciferase. The basal activity of the reporter was set to a value of 100. B) Quantitative ChIP assay of Myc and Omomyc binding to the *nucleolin* promoter region (Ncl; grey bars). Left: FLAG-c-Myc binding in 293T cells transfected with the *nucleolin* reporter pNucL14 together with FLAG-c-Myc and Omomyc expressing plasmids. Right: FLAG-Omomyc binding in 293T cells transfected with the *nucleolin* reporter together with FLAG-Omomyc and c-Myc expressing plasmids. A region of the *luciferase* coding sequence (Luc; black bars) was used as control. Bars represent the percentage of input DNA immunoprecipitated, after background subtraction. ChIP values are expressed as % of input DNA.

To investigate the mechanisms involved in gene regulation by Omomyc, we performed luciferase reporter and chromatin immunoprecipitation (ChIP) assays on two genes encoding the nucleolar protein nucleolin and the cyclin-dependent kinase inhibitor p21, respectively representing genuine and direct targets of Myc mediated activation and repression [51], [52]. c-Myc is able to activate *nucleolin* transcription via two highly conserved E-boxes in intron 1 [51]. To test the capability of Omomyc to inhibit Myc mediated transcriptional activation, we performed reporter assays in 293T cells transfected with the luciferase reporter plasmid pNucL14 [51] – containing the mouse *nucleolin* promoter, exon 1, intron 1 and the first 8 nt of exon 2 – together with different combinations of FLAG-c-Myc and Omomyc expression plasmids (Figure 5A). We found that Omomyc inhibited Myc mediated activation of the luciferase reporter in a dose dependent manner, while it did not affect its basal activity. To test whether the inhibition of *nucleolin* transactivation by Omomyc was due to a reduction of promoter binding, we measured the amount of Myc bound to the *nucleolin* promoter on 293T cells transfected with *nucleolin* reporter, FLAG-c-Myc and Omomyc expressing plasmids. Recovery of *nucleolin* DNA coprecipitated with Myc was quantified by real-time PCR, using primers located around the two E-boxes in *nucleolin* intron 1. We found that Omomyc caused a 40% reduction of the amount of promoter bound Myc (Figure 5B, left). Besides competing for Myc/Max association, Omomyc is able to form homodimers as well as Omomyc/Max dimers: only the latter bind E-boxes *in vitro* with high efficiency [9]. Therefore, Omomyc might affect Myc binding to DNA via two concurring mechanisms: inhibition of Myc/Max dimerization as well as direct competition for E-box binding. To assess the latter one, we measured the amount of Omomyc bound to the *nucleolin* promoter in cells transfected with c-Myc and FLAG-Omomyc expressing plasmids (Figure 5B, right). We found that Omomyc was specifically recruited to the E-box containing region in intron 1 and that it competed with c-Myc for binding to this region. Altogether, these data indicate that Omomyc can affect transactivation by sequestering Myc in complexes incapable of binding to E-boxes as well as by acting – presumably in association with Max – as competitive inhibitor of Myc/Max complexes for E-Box binding.

**Figure 6:**
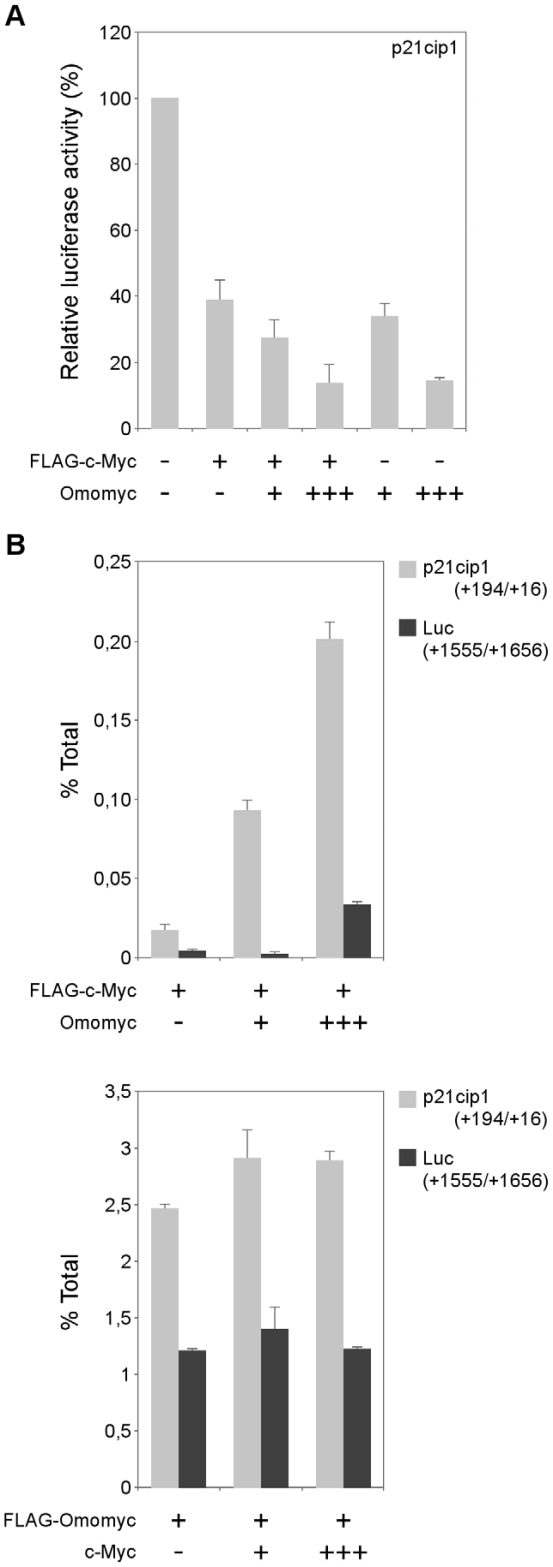
Omomyc promotes Myc binding and transrepression of the *p21* (*CDKN1*) promoter. A) Luciferase activity of the human *p21* promoter reporter plasmid – p21Cip1-Luc – transfected in 293T cells together with FLAG-c-Myc and Omomyc expressing plasmids. Data were normalized by cotransfection of pRL-TK Renilla luciferase. The basal activity of the reporter construct was set to a value of 100. B) Quantitative ChIP assay of Myc and Omomyc binding to the *p21* promoter region (p21cip1, grey bars). Top: FLAG-c-Myc binding in 293T cells transfected with the *p21* reporter p21Cip1-Luc together with FLAG-c-Myc and Omomyc expressing plasmids. Bottom: FLAG-Omomyc binding in 293T cells transfected with the *p21* reporter together with FLAG-Omomyc and c-Myc expressing plasmids. A region of the *luciferase* coding sequence (Luc; black bars) was used as control. Bars represent the percentage of input DNA immunoprecipitated, after background subtraction. ChIP values are expressed as % of input DNA.

To investigate how Omomyc may act to support Myc mediated repression, we conducted assays on the human *p21* promoter – specifically the sequence comprised between −194 and +16 from the transcriptional start site – on which Myc is recruited by the zinc finger protein Miz-1 [27], [52]. We performed reporter assays and found that luciferase expression driven by the full-length human *p21* promoter – p21Cip1-Luc [53] – was inhibited by c-Myc and that Omomyc was synergic with c-Myc (Figure 6A). Interestingly – even in the absence of a cotransfected c-Myc – Omomyc provoked *p21* promoter repression to levels similar to the ones caused by c-Myc. Therefore Omomyc is able to support *p21* promoter repression both in the presence and absence of an over-expressed c-Myc. To investigate whether this was associated to increased promoter binding, we performed ChIP assays on 293T cells transfected with the p21 reporter together with FLAG-Myc and increasing amount of Omomyc plasmid. We observed that Omomyc markedly increased c-Myc binding to the −194 to +16 region (Figure 6B, top). Interestingly, the reciprocal assay on cells transfected with FLAG-Omomyc and increasing amount of c-Myc plasmid indicated that Omomyc was capable of interacting with the same *p21* promoter region (Figure 6B, bottom). In this case, Myc overexpression did not compete with Omomyc binding. These data support the hypothesis that transrepression of Myc targets is sustained by Omomyc.

### Epigenetic modifications associated to Myc activity are affected by Omomyc

**Figure 7:**
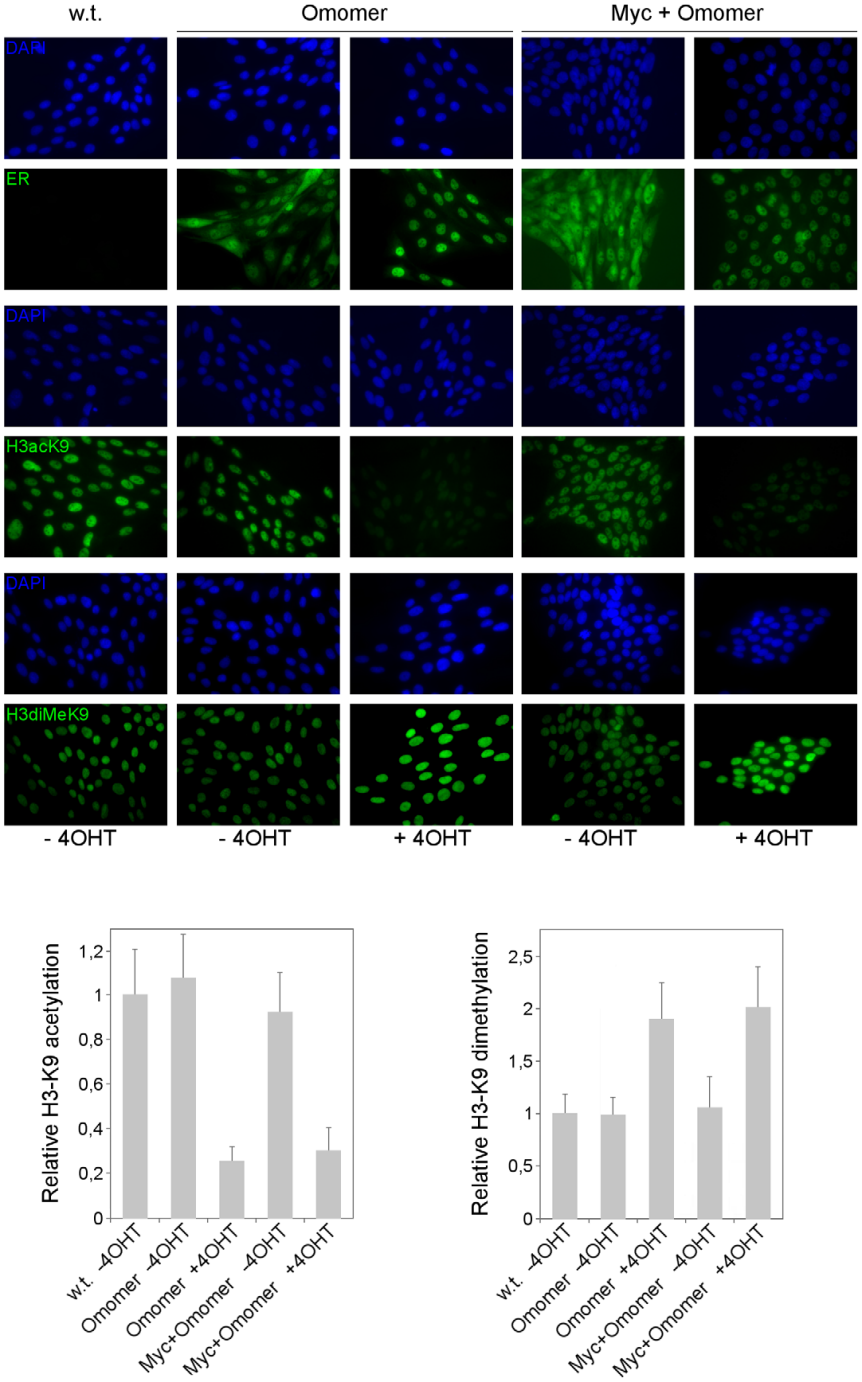
Omomyc influences – in an opposite way to c-Myc – the pattern of histone H3 lysine 9 acetylation and methylation, leading to decreased active and increased repressive chromatin marks. Immunofluorescence staining (in green) of ER (top), H3acK9 (middle) and H3diMeK9 (bottom panels) in parental (w.t.), Omomer, and c-Myc + Omomer expressing Rat1 fibroblasts [12] grown for 48 h with or without 4-OHT. DAPI staining (blue) was used for visualizing cell nuclei. The graphs below the immunofluorescence pictures display the quantitative analysis of H3AcK9 and H3diMeK9 staining. Values in the graphs represent fold change in histone H3 acetylation and dimethylation, relative to parental wild-type fibroblasts. Data were collected by densitometric analysis of nuclear fluorescence from three independent biological repeats.

**Figure 8:**
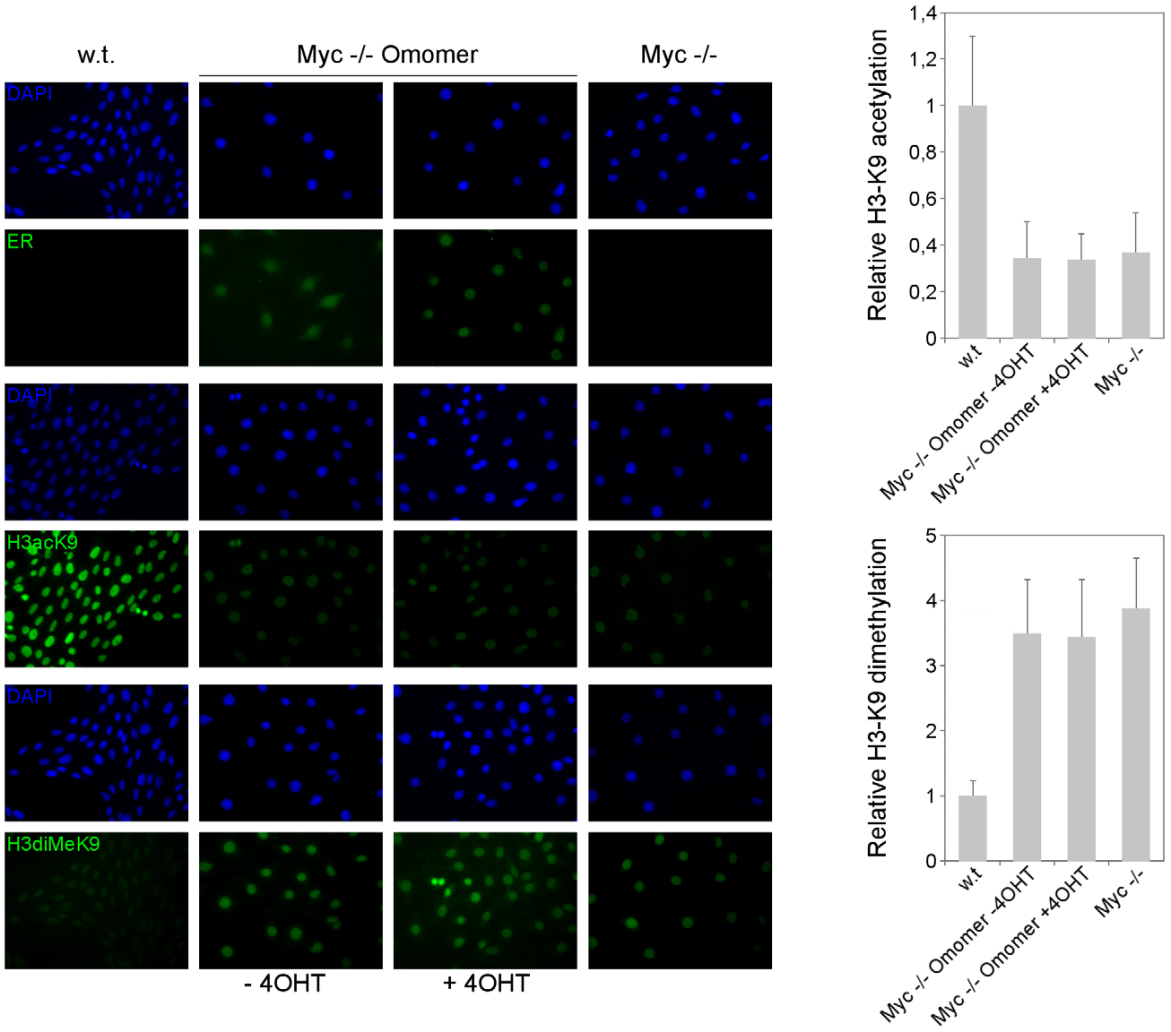
Omomyc does not influence the pattern of histone H3 lysine 9 acetylation and methylation in Myc null cells. Immunofluorescence staining (in green) of ER (top), H3acK9 (middle) and H3diMeK9 (bottom panels) in parental (w.t.), c-Myc null (Myc −/−), and c-Myc null expressing Omomer (Myc −/− Omomer) Rat1 fibroblasts [12] grown for 48 h with or without 4-OHT. DAPI staining (blue) was used for visualizing cell nuclei. The graphs at the right side of the immunofluorescence pictures display the quantitative analysis of H3AcK9 and H3diMeK9 staining: values represent fold change in histone H3 acetylation and dimethylation, relative to parental wild-type fibroblasts. Data were collected by densitometric analysis of nuclear fluorescence from three independent biological repeats.

Myc proteins are involved in the widespread maintenance of active chromatin. Disruption or downregulation of Myc expression leads to decreased H3 and H4 acetylation at selected histone residues accompanied by increased chromatin repressive marks, an effect that is reversed upon Myc reactivation [3], [38], [54], [55]. If Omomyc indeed targets Myc function, its overexpression should entail changes in the histone modifications associated with Myc activity. To test this hypothesis, we performed immunohistochemistry assays to detect global levels of histone H3 lysine 9 acetylation and dimethylation – representing marks of active and repressive chromatin – reported to be respectively decreased and increased upon Myc loss of function in neuronal progenitors and other cell types [54], [56]. N-Myc genomic binding is strongly linked to H3AcK9 while loss of N-Myc decreases the pool of this active chromatin mark [54]; 90% to 95% of the H3AcK9 mark in a human neuroblastoma cell line was reported to be N-Myc dependent [55]. We assessed levels of H3acK9 and H3diMeK9 in parental Rat1 cells (w. t.), in Rat1 cells harboring an inducible Omomyc (Omomer) and in Rat1 cells harboring the inducible Omomyc and an overexpressed c-Myc (Myc+Omomer) (Figure 7). For a comparison, H3K9 acetylation and dimethylation was also measured in Myc null Rat1 cells (Myc −/−) and Myc null cells harboring the tamoxifen inducible Omomyc (Myc −/− Omomer) (Figure 8). We found that wild type Rat1 cells displayed significant staining of H3acK9 and faint staining of H3diMeK9. Treatment with tamoxifen triggered Omomer translocation to the nucleus, as expected (Figures 7, top panels), which was accompanied by a dramatic reduction of the H3acK9 signal, not affected by a concomitant Myc overexpression (Figure 7, middle panels). Omomyc caused an approximately four-fold reduction in H3acK9 according to the densitometric analysis (Figure 7). Myc null Rat1 cells displayed the opposite pattern to parental cells with significant H3diMeK9 staining and a quasi-complete loss of H3acK9 staining, similarly to what was reported for N-Myc null neuroprogenitor cells [54]. This pattern was unaffected upon Omomer induction (Figure 8). Of note, H3acK9 levels in Omomyc overexpressing cells were even lower than observed in Myc null cells, possibly due to compensatory events that occurred in the latter. The H3K9 hypoacetylation observed upon Omomer induction in the Omomer and Myc+Omomer expressing Rat1 cells was associated with a two-fold enhancement in dimethylation of the same histone residue (Figure 7, bottom panels). In Myc null fibroblasts, instead, Omomer induction did not affect the levels of H3K9 dimethylation (Figure 8, bottom panels). The data demonstrate that Omomyc impacts histone H3K9 acetylation and methylation in an opposite way to Myc, leading to decreased active and increased repressive chromatin marks.

### Omomyc affects proliferation and survival of cells in culture

**Figure 9:**
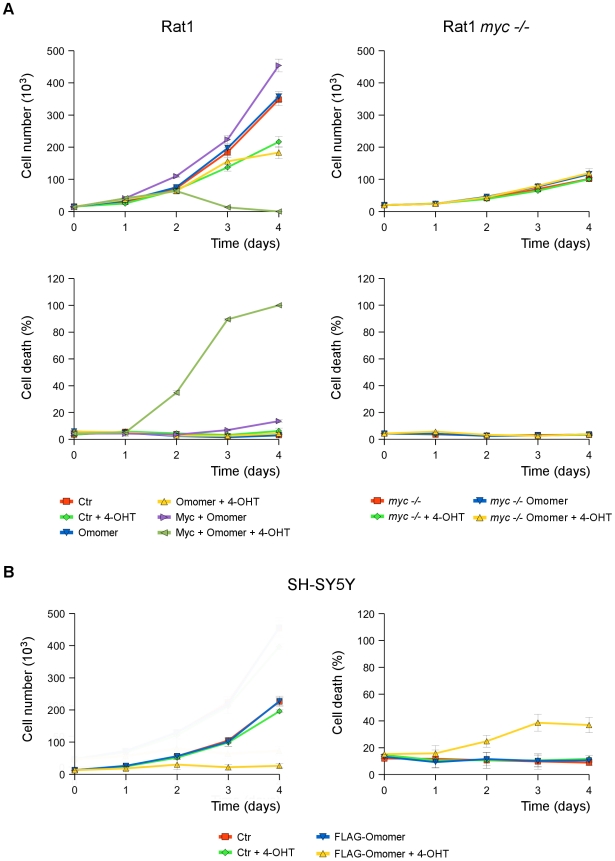
Effect of Omomyc ectopic expression on proliferation and death of Rat1 and SH-SY5Y cells. A) **Omomyc ability to hamper growth and induce apoptosis of Rat1 fibroblasts is Myc dependent.** Growth curves (top panels) and percentage of death cells (bottom panels) of wild type (Rat1, left panels) and Myc null (Rat1 *myc −/−*; right panels) fibroblasts over expressing – or not (Ctr) – Omomer or c-Myc and Omomer (Myc+Omomer) [12]. B) **Omomyc is able to reduce growth and promote apoptosis of the human neuroblastoma cell line SH-SY5Y.** Growth curve (left) and percentage of cell death (right) of SH-SY5Y human neuroblastoma infected with control or Omomer (FLAG-Omomer) expressing lentiviruses. Cells (1.5×10^5^) were plated in multi-well plates in presence and absence of 4-OHT. Proliferation and death were assayed daily by cell count and vital staining with trypan blue. Data represent three independent biological repeats.

Omomyc was shown to strongly potentiate Myc induced apoptosis in murine myoblasts [10], and to slow growth and trigger death of the tumor cells while sparing the surrounding normal tissue in a lung adenocarcinoma model [16]. To further assess its effects on cell growth and death, we determined proliferation and survival of Rat1 fibroblasts constitutively expressing – or not – c-Myc and Omomer [10] (Figure 9A). In the absence of tamoxifen, Rat1 cells expressing or not c-Myc and Omomer had similar growth and death rate. Rat1-control and Rat1-Omomer cells behaved similarly also upon tamoxifen treatment, which modestly slowed down proliferation of both. Rat1 cells co-expressing c-Myc and Omomer instead behaved differently from the other two cell types in the presence of tamoxifen: initially they grew similarly, but after two days their proliferation stopped two due to massive cell death (Figure 9A). Therefore, Omomyc appeared to only affect survival of Rat1 cells over-expressing c-Myc, in agreement with the hypothesis that activation of the cell death pathway requires Myc over-expression [57]. Finally, we measured proliferation and death of SH-SY5Y human neuroblastoma cells infected with Omomer producing or control lentiviruses (Figure 9B). Switching on Omomyc activity by tamoxifen in the neuroblastoma cells quickly inhibited growth and triggered death, whereas tamoxifen had no effect on cells infected with the control virus (Figure 9B). Omomer expression in lentivirus infected neuroblastoma cells was stronger than in Rat1-Omomer cells (not shown), suggesting that the greater sensitivity of the neuroblastoma cell line – as compared to Rat1 cells – to the action of Omomyc might reflect its higher expression level.

## Discussion

The interest in Omomyc derives from its outstanding anti-tumor activity, unparalleled by other Myc inhibitory treatments [16]. This molecule selectively affects the Myc protein interaction network. On the basis of our results, we propose that Omomyc acts like an edge-specific perturbation of the network that produces opposite effects on the two arms of Myc activity: transactivation and transrepression of gene transcription. Edgetic perturbations of a protein network confer distinct functional consequences from node removal [58], achieved by technologies like gene knockout or RNAi. The finding that Omomyc can bind N-Myc as well as c-Myc and, presumably, all Myc family proteins suggests that it may be able to prevent cells from eluding inhibition of a single Myc protein by upregulating another Myc family member. Omomyc – by inhibiting Myc interaction with Max – suppresses binding to E-boxes and transactivation by Myc, whereas – by allowing Myc interaction with Miz-1 – it favors binding to promoters of repressed targets like p21 and transrepression. As a result, the Myc network action is channeled to transrepression. This conclusion is supported by the changes – in transcriptional response to serum stimulation and epigenetic marks – observed in fibroblast cells ectopically expressing Omomyc. Upon serum stimulation, many more genes were downregulated in the presence of Omomyc than in its absence, and these genes were enriched in genuine, direct Myc targets. Our data also suggest that Omomyc by itself may directly contribute to downregulation of Myc repressed targets by associating with Miz-1. It is presently unclear whether the enhancement of transrepression by Omomyc may be solely explained via Miz-1, as other transcription factors as well – e.g. NFY and SP1 – have been reported to interact with Myc to promote promoter binding and transrepression [4]. Further experiments – like the design of Omomyc variants that hamper the interaction with Miz-1 – will be required to assess these points. Another contribution to the pro-repressive action is given by the decrease of activating and the increase of repressive chromatin marks – respectively H3K9 and H3diMeK9 – in the presence of Omomyc. Myc elicits changes in the histone acetylation and methylation patterns by two distinct mechanisms [54], both of which can be affected by Omomyc. Specifically, Myc was shown to recruit a number of proteins with chromatin modification activity – histone acetyltransferases and methyltransferases, a H3-specific kinase, a histone deacetylase, a histone H3-K4 dimethylase [38], [59] – and to activate target genes encoding chromatin modification enzymes like the histone acetyltransferase GCN5 [54] and the Lysine-specific demethylase 5C [38]. While the upregulation of these target genes by Myc is presumably hampered by Omomyc, the direct influence of Omomyc on the multiple interactions of Myc with epigenetic modulatory proteins remains to be determined. Omomyc strongly affected proliferation and apoptosis in two cell lines – immortalized Rat fibroblasts and a human neuroblastoma – in a way that was correlated to its expression level.

Clearly, Omomyc itself is not a therapeutic agent, but serves as a tool that models the efficacy of future strategies that interfere with Myc function in tumorigenesis [60]. Our study has implications for the design of inhibitors that would target Myc for cancer therapy. The development of small-molecule inhibitors of protein-protein interactions is challenging, but significant progress is being made and bHLH-ZIP proteins are considered promising targets [13]. Most attempts to target Myc for cancer therapy focused on inhibiting the Myc/Max association and preventing Myc binding to E-boxes [12], [13], [61], [62]. While this is clearly a crucial issue, our data indicate that this is likely not to be enough and that the repressing arm of Myc should be taken into account. It may be equally important to safeguard at least some aspects of the Myc transrepressive arm while disabling the activating one. This conclusion is in agreement with evidence indicating that transrepression has an important role in apoptosis, senescence and tumorigenesis. The particular type of perturbation introduced by Omomyc in the Myc interactome may turn the transrepressive arm in a powerful tumor suppressor, promoting cancer cell death.

## Materials and Methods

### Cell culture

Rat1 fibroblasts expressing Omomer and c-Myc + Omomer, and c-Myc null Rat1 (*myc −/−*) fibroblasts expressing Omomer were described previously [10]. Rat1 (*myc −/−*) fibroblasts were obtained by J. Sedivy [63]. Rat1 and HEK 293T cells [64] were cultured in complete DMEM medium and SH-SY5Y neuroblastoma cells [65] in DMEM F12, supplemented with 10% foetal calf serum (EuroClone) and 1% penicillin/streptomycin at 37°C in 5% CO_2_. For Omomer induction, cells were treated with 4-OHT (Sigma), added to the culture medium at a final concentration of 5×10^−7^ M. For serum induction, cells were grown for 48 h in 0.1% serum (serum-starvation) and switched to media with 10% serum in the absence or presence of 4-OHT (added 4 h before switching to 10% serum). Transfections were performed using Lipofectamine 2000 (Invitrogen).

### Plasmids

Omomyc and Omomer expressing plasmids (pCS-Omomyc and pBP-Omomer) were described previously [9], [10]. c-Myc, FLAG-c-Myc and FLAG-N-Myc expressing vectors (cβS-c-Myc, cβSFLAG-c-Myc and cβSFLAG-N-Myc) were from M. Cole; the Miz-1 expressing vector (pCMV-Miz-1) was from M. Eilers and the Hif-1α vector (pCMV- Hif-1α) was obtainded from A. Levi. GST-HEB and GST-ID1 expressing plasmids were previously described [65]; the GST-MAD plasmid was from R. Eisenman and the His-Max expressing plasmid was from L. Lania. Luciferase reporters of the nucleolin and p21 promoters (pNucL14 and p21Cip1-Luc) were from B. Amati. The cβSFLAG-Omomyc expression plasmid and the Tween lentiviral vector [66] expressing FLAG-Omomer were assembled according to standard procedures by means of the following oligonucleotide primers:

cβSFLAG-Omomyc: 5′-GGCCCCCGGGACCGAGGAGAATGTCAAGAGG-3′ (forward) and 5′-GGCCAAGCTTTTACGCACAAGAGTTCCGTAG-3′ (reverse).

Tween-FLAG-Omomer 5′-GGCCGTCGACATGGACTACAAGGACGATGAT-3′ (forward) and 5′-GGCCGATATCACTAGTAGGAGCTCTCAGAT-3′ (reverse).

### Pull-down assay

Pull-down assays with GST-linked proteins (Heb, Id1, Max, MAD) were performed as previously described [65].

### Coimmunoprecipitation

pBP-Omomer, pCMV-Miz-1, cβSFLAG-c-Myc, cβSFLAG-N-Myc, pJ4Ω-Max, pCMV- Hif-1α and cβSFLAG-Omomyc plasmids were transfected into 293T cells. After 2 days, cells were collected and processed as previously described [65].

### Immunoblotting

Western blot analysis was performed as described previously [65], using the following antibodies and reagents: ER (MC20, Santa Cruz Biotechnology; 1∶1000 dilution), Miz-1 (H190, Santa Cruz Biotechnology; 1∶1000), Hif-1α (clone-54, BD Transduction Laboratories; 1∶1000), Max (c-124, Santa Cruz Biotechnology; 1∶1000 dilution), and FLAG (M2, Sigma; 1∶2500) antibodies; peroxidase conjugated rabbit anti-mouse IgG (Chemicon; 1∶10000), peroxidase conjugated protein A (Sigma; 1∶10000). Densitometric analysis of immunoblots was done by the ImageJ program.

### Immunofluorescence

Cells grown on glass chamber slides were washed in PBS, fixed for 10 min in 4% paraformaldehyde, treated for 5 min with 0.2% Triton X-100 and processed for immunofluorescence as previously described [65]. The following antibodies were used: H3acK9 (04-1003, Millipore; 1∶200 dilution), H3diMeK9 (07-212, Millipore; 1∶200), ER (MC20, Santa Cruz; 1∶200), c-Myc (N-262, Santa Cruz; 1∶200), Miz-1 (H190, Santa Cruz; 1∶200), FLAG (M2, Sigma Aldrich; 1∶1000), FITC conjugated rabbit anti-mouse IgG (Chemicon; 1∶1000) and Rhodamine conjugated goat anti-rabbit IgG (Chemicon; 1∶1000). DAPI (200 ng/ml) was used for staining of nuclei. Images were acquired by a Nikon fluorescence light microscope, through NIS-Elements 3.1 software. Fluorescence intensity was determined using ImageJ software.

### mRNA expression profiling and data analysis

Total RNA was extracted with TRIzol reagent (GIBCO-BRL) according to the manufacturer's protocol. RNA was checked for quantity, purity, and integrity by gel electrophoresis and UV spectrophotometric measurements: 10 µg of total RNA were used as starting material for preparing cDNA. Preparation of biotinylated target RNA – synthesis and cleanup of double-stranded cDNA followed by synthesis, cleanup and fragmentation of biotin-labeled cRNA – was done according to the instructions provided in the Affymetrix GeneChip Expression Analysis manual. Each biotin-labelled sample was hybridized onto a GeneChip® Rat Genome Probe Array RG-U34A from Affymetrix for 16 h at 45°C in a GeneChip Hybridization Oven - according to the manufacturer's recommendation. The expression probe arrays were washed and stained through a GeneChip Fluidics Station 400 according to the Affymetrix GeneChip Expression Analysis manual. The probe arrays were scanned using an HP Gene Array Scanner, controlled by the GeneChip software. Data were processed with the GeneChip Microarray Analysis Suite version 4.0 software (MAS 4, Affymetrix) with default analysis settings; genes were sorted according to robust analysis rules. The Rat1-control cell (time 0) sample was taken as reference for calculating relative expression values as Fold Change (www.affymetrix.com/support/technical/whitepapers.affx: Statistical Algorithms Description Document). Fold Changes were computed for Rat1-control cells at 90′ following serum stimulation as well as for the Rat1-Omomer cells at 0 and 90 minutes time points. Data were filtered by setting a Fold Change threshold of +3 for the activated genes and −3 for the repressed ones. All data are MIAME compliant and the raw data were deposited in the GEO database, with accession number GSE25039. Correspondence Analysis (CA) was also performed on the same data, via routine present in the SAS (Statistical Analysis Software) package. The results obtained through CA were compared with those of the selection through robust analysis via MAS 4 software. We observed that 89% of genes identified by the manual procedure as decreased at 90 min in the presence of Omomyc were also detected by the CA. The same comparison on genes increased by the induction of Omomyc at 90 min gave again a remarkable fit between the two methods, with about 87% genes in common.

To determine overlap among genes downregulated by Omomyc and Myc target genes, we crossed the Omomyc gene list with those of the Myc cancer gene database and genes whose promoters were reported to be occupied by Myc in ES cells [36], [37]. 623 of the genes listed in the Myc Cancer Gene database and 984 of those listed as Myc bound targets in ES cells were present in the U34A array; genes not represented in the U34A microarray were excluded from the analysis. Omomyc-associated genes were annotated using Panther software (http://www.pantherdb.org/). Genes not assigned to a biological process (biological process unclassified) were not included in the figures.

### Luciferase reporter assay

293T cells, seeded in 24-well tissue culture plates, were transfected with *nucleolin* or *p21* reporters – respectively pNucL14 and p21Cip1-Luc – driving expression of firefly luciferase, the Renilla luciferase reporter – pRL-TK from Promega – and combinations of Myc (cβSFLAG-c-Myc) and Omomyc (pCS-Omomyc) expressing plasmids. Cells were lysed 48 h later and luciferase activities were measured by the Dual Luciferase reporter assay (Promega).

### Quantitative Chromatin Immunoprecipitation

293T cells, transfected with pNucL14 or p21Cip1-Luc, together with different combinations of cβSFLAG-c-Myc, cβSFLAG-Omomyc, pCS c-Myc and pCS Omomyc DNA, were processed as described in [3]. The FLAG M2 antibody (Sigma) was used for immunoprecipitation. Real-time PCR was performed with 6 µL of DNA per reaction in iTAQ™ SYBR Green Supermix (BioRad). Accumulation of fluorescent products was monitored using a GeneAmp 9700 Sequence Detector (ABI). Each PCR reaction generated only the expected specific amplicon, as shown by the melting-temperature profiles of final products. No PCR products were observed in the absence of template. The indicated regions of *nucleolin* promoter and *luciferase* coding sequence – used as control – were amplified by qPCR. Background subtraction was calculated from the amount of reporter construct immunoprecipitated by anti-FLAG antibody in absence of FLAG-c-Myc and from the no-antibody samples.

Primer sequences. *Nucleolin*: (forward) 5′-CCCTTTCCGGGGTACTACAG-3′, (reverse) 5′-GGAAGAGAGGGCCAACCTTA-3′; *p21*: (forward) 5′-ACCGGCTGGCCTGCTGGAACT-3′, (reverse) 5′-TCTGCCGCCGCTCTCTCA CCT-3′; firefly *luciferase* coding sequence: (forward) 5′-GGAAAGACGATGACGGAAAA-3′, (reverse) 5′-CGGTACTTCGTCCACAAACA-3′

### Gene expression analysis by Real-Time PCR

Real Time PCR for gene expression analysis was performed on cDNA retro-transcribed (kit from BIO-RAD) from total RNA isolated by TRIzol reagent. PCR reactions were performed by iTaq SYBR Green Supermix With ROX kit (BIO-RAD). Accumulation of fluorescent products was monitored using a GeneAmp 9700 Sequence Detector (ABI). At least two independent amplifications were performed for each probe, with triplicate samples. Cycle threshold values were determined by automated analysis. Each PCR reaction generated only the expected specific amplicon, as shown by the melting-temperature profiles of final products. No PCR products were observed in the absence of template.

*Gadd45a*: (forward) 5′-CAGAGCAGAAGATCGAAAGGA-3′, (reverse) 5′-GACTCCGAGCCTTGCTGA-3′; *Akt1* (forward) 5′-AACGACGTAGCCATTGTGAA-3′, (reverse) 5′-CCATCATTCTTGAGGAGGAAGT-3′; *Ccnd1* (forward) 5′-GCACAACGCACTTTCTTTCC-3′, (reverse) 5′-TCCAGAAGGGCTTCAATCTG-3′; *Erbb2* (forward) 5′-AGCTCAGAGACCTGCTTTGG-3′, (reverse) 5′-AGGAGGACGAGTCCTTGTAGTG-3′; *Eif3s9* (forward) 5′-ACTGGCCGCTATGTGGTTAC-3′, (reverse) 5′-CAGCCAATAAGCATTGTCCA-3′; *Mxi1* (forward) 5′-CGGATGATCAACGTGCAG-3′, (reverse) 5′-GCGTAGCCATGTTCACACTC-3′; *Pgk1* (forward) 5′-CCAGATAACGAATAACCAAAGGA-3′, (reverse) 5′-GACTTGGCTCCATTGTCCA-3′

### *In vitro* growth curves

To determine growth curves, 1.5×10^4^ cells were plated in triplicate in 12-well plates. From the next day, cells were trypsinized and counted daily with a haemocytometer for four days. Cell death was assessed by trypan blue staining.

## Supporting Information

Table S1 List of genes organized into two pages (UP Omomer and DOWN Omomer) according to whether they are activated or repressed by Omomyc upon 90 min of serum stimulation of Rat1 cells. Genes were selected according to the criteria described in Results. Gene ID, gene symbol and name are shown, together with the probe set ID of the Affymetrix U34A array. The last two columns denote, respectively, whether genes are listed as upregulated (UP) or downregulated (DOWN) by Myc in the Myc target gene database and whether their promoters are listed as Myc bound promoters in either one of two studies performed in embryonic stem cells [36], [37].
(XLS)
